# Exploring the Processing Potential of Polylactic Acid, Polyhydroxyalkanoate, and Poly(butylene succinate-*co*-adipate) Binary and Ternary Blends

**DOI:** 10.3390/polym16162288

**Published:** 2024-08-13

**Authors:** Alisa Sabalina, Sergejs Gaidukovs, Arturs Aunins, Anda Gromova, Gerda Gaidukova, Liga Orlova, Oskars Platnieks

**Affiliations:** 1 Institute of Chemistry and Chemical Technology, Faculty of Natural Sciences and Technology, Riga Technical University, P. Valdena 3, LV-1048 Riga, Latviaanda.gromova@rtu.lv (A.G.); oskars.platnieks_1@rtu.lv (O.P.); 2 Institute of Materials and Surface Engineering, Faculty of Natural Sciences and Technology, Riga Technical University, P. Valdena 3, LV-1048 Riga, Latvia

**Keywords:** thermal properties, biopolyester, biodegradable, calorimetric properties, rheological properties

## Abstract

Biodegradable and bio-based polymers, including polyhydroxyalkanoate (PHA), polylactic acid (PLA), and poly(butylene succinate-*co*-adipate) (PBSA), stand out as sustainable alternatives to traditional petroleum-based plastics for a wide range of consumer applications. Studying binary and ternary blends is essential to exploring the synergistic combinations and efficiencies of three distinct biopolyesters. A comprehensive evaluation of melt-extruded binary and ternary polymer blends of PHA, PLA, and PBSA was conducted. Scanning electron microscopy (SEM) analyses revealed a heterogeneous morphology characteristic of immiscible blends, with a predominant spherical inclusion morphology observed in the majority of the blends. An increased PBSA concentration led to an elevation in melt viscosity and elasticity across both ternary and binary blends. An increased PHA content reduced the viscosity, along with both storage and loss moduli in the blends. Moreover, a rise in PHA concentration within the blends led to increased crystallinity, albeit with a noticeable reduction in the crystallization temperature of PHA. PLA retained amorphous structure in the blends. The resultant bio-based blends manifested enhanced rheological and calorimetric traits, divergent from their pure polymer counterparts, highlighting the potential for optimizing material properties through strategic formulation adjustments.

## 1. Introduction

Growing environmental concerns, notably those about single-use plastics and the depletion of non-renewable resources, have driven the scientific community to seek sustainable alternatives to petroleum-based polymers in the packaging sector [1]. Annual plastics production is more than 350 million tons, which is increasing at a yearly rate of 4% [2]. In 2022, bio-based polymers account for about 1% of the total production volume of fossil-based materials. The global bio-based polymers market is expected to grow by 14% between 2022 and 2027 [3,4]. Commodity plastic alternatives that are bio-based and/or biodegradable are bioplastics. Bio-based plastics are materials derived from biomass resources like corn, starch, and wood and produced through a combination of microbial metabolic activities and industrial synthesis [5,6,7]. These polymers provide a sustainable alternative to petroleum-based polymers and can play a pivotal role in reducing CO_2_ emissions, managing plastic waste, and conserving fossil fuels [8,9,10].

The established market for fossil-based polymers offers a diverse range of grades tailored for specific engineering solutions. In contrast, the market for bio-based and biodegradable polymers and the available grades is small and limited [11,12]. Also, the chemical composition and molecular weight play an essential role in the biodegradation rates of aliphatic polyesters [13]. Polyhydroxyalkanoates (PHA) are a biodegradable polyester group produced by microorganisms. PHA has adjustable mechanical properties based on the targeted structure. However, its poor processability, relatively high price, and limited commercial availability restricts its use in various applications [14,15]. Polylactic acid (PLA) is one of the most widely used bio-based polyesters. PLA has good processability and is suitable for many applications, but can be brittle and lack toughness [16,17,18]. Poly(butylene succinate) (PBS) and its copolymers, like poly(butylene succinate-*co*-adipate) (PBSA), are partially bio-based flexible polyesters with good processability, but their poor barrier properties have been reported in some sources [19,20]. PBSA is flexible at room temperature, and its melting point (80–90 °C) is low compared to other widely used bioplastics such as PLA [19,20]. In a bid to harness the best properties of bio-based polymers and mitigate their limitations, blending has emerged as a compelling strategy [1,11]. While synthesizing entirely new polymers can be resource-intensive, mixing two or even three distinct polymers can effectively fine-tune their properties [21,22,23,24].

This approach has resulted in binary blends, such as the one reported by Aliotta et al., where a mere 20 wt.% of poly(butylene succinate-*co*-adipate) (PBSA) amplified the elongation at break in PBSA/poly(lactic acid) (PLA) blends exponentially [25]. Similarly, Righetti et al.’s work on poly(3-hydroxybutyrate) (PHB)/poly(butylene succinate) (PBS) and PHB/PBSA blends highlighted how the ductility of these blends could be enhanced with the addition of PBS and PBSA [26]. Yang et al. reported that molten droplets of PBSA acted as nucleating agents for plasticized PLA [27]. Olejnik et al. reported that incorporating PHB into the PHB/PLA blend increases the melt volume flow rate and reduces the glass transition temperature [28]. D’Anna et al. noted the typical rheological behavior of immiscible PLA/PHB blends, manifesting as peaks at low to intermediate frequencies, attributed to the relaxation of the dispersed phase in droplet form [29]. Also, the authors reported that miscibility between PLA and PHB depended on the molecular weight of the components [30]. PLA with a low molecular weight (M_w_ < 18,000) is miscible with PHB, while the high-molecular-weight PLA (M_w_ > 18,000) showed two separated phases with PHB [31,32].

Significant interest in developing ternary or multicomponent systems grows annually [33]. Arrigo et al. reported that the ternary PLA/PBS/poly(3-hydroxybutyrate-*co*-3-hydroxyhexanoate) (PHBHH) blends demonstrated enhancements in toughness and impact strength compared to neat PLA without significantly reducing stiffness and tensile strength [10]. Ternary blends containing PLA, poly(3-hydroxybutyrate-*co*-3-hydroxyvalerate) (PHBV), and at least 40% polypropylene carbonate (PPC) provide balanced thermal and mechanical properties, which are necessary for high-performance applications [21]. The authors reported that the PLA/PHBV/PPC optimal ratio was 20/40/40, which yields elongation at a break of 215% and elastic modulus of 2.7 GPa. Zhang et al.’s research on biodegradable ternary blends comprising PLA, PHBV, and PBS emphasized that the presence of PHBV augmented the crystallization of PLA and PBS [33]. Conversely, the crystallization of PHBV was restricted by the PLA and PBS phases.

Moreover, blends like PHA, PLA, and PBSA encounter challenges due to their phase-separated morphology and the inability of the components to mix well, leading to compromised material properties [34,35,36]. The blending process, the viscosity and molecular weight ratio between the polymers, crystallization behaviors, and the proportion of each polymer significantly influence the blend’s structure [37]. Wang et al. have shown that the presence of PBS crystallites can lower the temperatures at which PLA undergoes glass transition, melting, and cold crystallization, demonstrating the impact of heterogeneous nucleation [34]. Arrigo et al., using SEM microscopy, analyzed the PLA/PBS/poly (3-hydroxybutyrate-*co*-3-hydroxyhexanoate) (PHBHH) composite structure [10]. They found that formulations with a minor PBS component—specifically, the 70/15/15 and 50/20/30 ratios—exhibited a distinct droplet–matrix phase morphology, illustrating the polymer’s inability to form miscible blends.

This research delves into the rheological and calorimetric properties of both binary and ternary blends, specifically involving polyhydroxyalkanoate (PHA), PLA, and PBSA. Innovative engineering blends can be designed by examining their flow behaviors under varying shear conditions and assessing their thermal transitions. These new compositions aim to capitalize on the strengths of each polymer while minimizing their shortcomings. A comprehensive examination of these blends is timely and essential as the biopolymer landscape continues to evolve, driven by ecological imperatives and technological advancements.

## 2. Materials and Methods

### 2.1. Materials

Poly(butylene succinate-*co*-adipate) (PBSA) FD92PM was purchased from PTT MCC Biochem Co., Ltd. (Bangkok, Thailand). PBSA is a semi-crystalline thermoplastic polyester with a 1.24 g/cm^3^ density and a melt flow index (MFI) of 4 g/10 min (measured at 2.16 kg and 190 °C). It is fully biodegradable and demonstrates partial bio-based composition, comprising 20–50% bio-derived content (DIN certification 8C083). Polyhydroxyalkanoate (PHA) PHI002 was purchased from Natureplast^®^ (Caen, France). It is a semi-crystalline thermoplastic polyester with a melting temperature of 170 to 176 °C, a melt flow index of 5–10 g/10 min (measured at 2.16 kg and 190 °C), and a 1.23 g/cm^3^ density. PHA is certified to be more than 90% biobased (ASTM D6866 standard) [38] and industrially compostable following the ASTM D6400 standard [39]. Polylactic acid (PLA) with the trademark Ingeo™ and grade 6201D, manufactured by NatureWorks^®^ LLC (Minneapolis, MN, USA), was acquired from a local distributor. PLA 6201D is a 100% bio-based and compostable resin designed explicitly for fiber production. It has a melting temperature of 170 °C, a melt flow index of 15–30 g/10 min (measured at 210 °C, according to ASTM D1238) [40], and a 1.24 g/cm^3^ density.

### 2.2. Blend Preparation

The polymer granules underwent desiccation within a vacuum furnace (J.P. Selecta, Barcelona, Spain) at 40 °C under a controlled pressure regime ranging between 5 and 20 mbar for 24 h. Polymer blends were melt-mixed employing the Thermo Electron PRISM TSE 16 TC bench-top twin-screw extruder (Waltham, MA, USA). The formulations of the blends used are presented in Table 1. During the extrusion process, the barrel temperatures were set as follows: 160 °C in the feeding zone, and 165 °C, 170 °C, 175 °C, and 180 °C at the die. The screws were set at a rotation speed of 24 rpm. The resultant extruded strands, approximately 3 mm in diameter, underwent subsequent cooling via immersion in a water bath and then pelletization to a length of 2 mm using Thermo Electron PRISM VARICUT 1 equipment. After pelletization, the obtained pellets were subjected to an additional desiccation step in the vacuum furnace at 40 °C (5–20 mbar) for 24 h. The desiccated pellets were hermetically enclosed within impermeable plastic pouches.

### 2.3. Sample Preparation

Polymer films with a thickness of 0.85 mm were fabricated for rheology tests. They were made by compression molding using a Carver 4386 hydraulic press at 130 °C for PBS, 170 °C for PHA and PHA/PBS blends, and 180 °C for the other blends. They underwent preheating for 2 min, compression for 2 min (3 metric tons), and rapid cooling between two stainless steel plates with a total mass of 20 kg. Prepared pellets were used for differential scanning calorimetry (DSC) tests.

For SEM tests, rod-shaped samples were fabricated using a BOY 25E injection molding machine. The thermal profile applied for the mixtures in the extrusion area was as follows: 160 °C in the feeding zone, followed by increments to 165 °C, 170 °C, 175 °C, and 180 °C at the die. It is important to note that the thermal profile differed for the PBS and PHA/PBS mixtures. Expressly, the thermal profile for PBS was set at 105 °C, 110 °C, 115 °C, 120 °C, and 125 °C at the die. Meanwhile, the thermal profile for PHA/PBS mixtures comprised temperatures of 153 °C in the feeding zone, followed by 158 °C, 163 °C, 168 °C, and 173 °C. The blends were pressed into a mold at a temperature of 40 °C for the blends, PHA, and PLA, while a mold temperature of 20 °C was used for the PBS. During this process, a filling rate of 20 mm/s was applied with a pressure of 90 bar. The formed samples were then cooled within the mold for a duration of 50 s.

### 2.4. Testing Methods

DSC measurements were conducted on the Mettler Toledo DSC-1 analyzer [41]. The measurements were made under a nitrogen atmosphere and consisted of heating, cooling, and a second heating in the temperature range of −50–220 °C, using the neat polymer and blend pellets. The heating and cooling rate was 10 °C/min, and the average weight of samples was approximately 10 mg. The crystallinity of binary and ternary systems was calculated by using Equation (1), as follows:

(1) $$\chi \%=\frac{\Delta H}{\Delta H^{0}\left(1-W_{f}\right)}$$

where $W_{f}$ is the weight fraction of the second and third components (PHA, PLA, and PBSA) in the blend, ΔH is the experimental crystallization enthalpy (J/g) of the polymer in the blend, and $\Delta H^{0}$ is the melting enthalpy for 100% crystalline polymer taken from the literature. The value of $\Delta H_{PHA}^{0}$ is 146 J/g, $\Delta H_{PLA}^{0}$ is 93 J/g, and $\Delta H_{PBSA}^{0}$ is 110 J/g [42,43,44,45].

The Anton Paar Smart-Pave 102 (Graz, Austria) measured the polymer melt rheology. A parallel plate measuring system with a 25 mm diameter and a fixed temperature of 180 °C was used. The first oscillatory measurements were determined at torques ranging from 0.2 to 60 N∙m and were used to investigate the linear viscoelastic properties. The second oscillatory measurement was registered at an angular frequency range of 0.1–628 rad/s and a fixed strain amplitude of 5%. The measurements shown are the average of three repeat samples.

Scanning electron microscopy (SEM) was performed using a FEI Nova NanoSEM 650 (Eindhoven, The Netherlands) Schottky field emission scanning electron microscope (FESEM). Fractured sample surfaces were prepared through immersion in liquid nitrogen and were mounted on electrically conductive double-sided carbon tape for imaging. An acceleration voltage of 10 kV was used for image generation.

## 3. Results

### 3.1. Rheology

**Figure 1:**
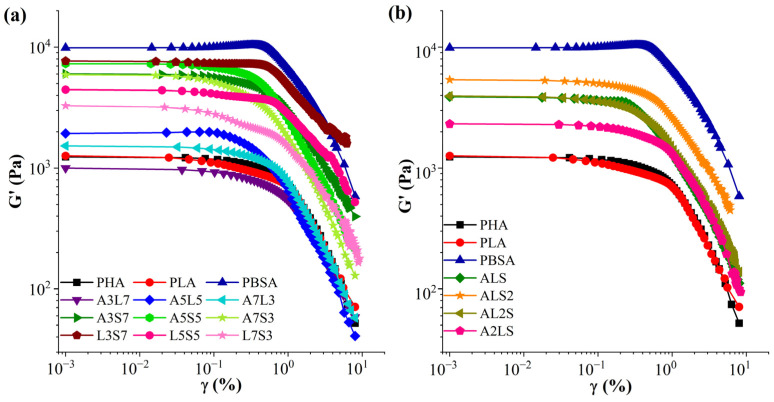
Strain (γ) dependence of storage moduli (G′) of (**a**) binary PHA/PLA, PHA/PBSA, and PLA/PBSA and (**b**) ternary PHA/PLA/PBSA blends at 180 °C.

Dynamic strain sweep measurements were performed to determine the linear viscoelastic limits of binary and ternary PHA, PLA, and PBSA blends. Storage moduli (G′) as a function of strain (γ) for binary and ternary blends are shown in Figure 1. The modulus values exhibited a range spanning an order of magnitude, with PLA and PHA positioned at the lower end and PBSA at the higher end. The blends generally displayed modulus values intermediate between neat polymers, albeit with minor deviations from this pattern. Increasing the concentration of PBSA in binary and ternary blends increased the storage moduli at lower strains. The viscoelastic behavior of the blends was similar to that of the neat polymers, with the linear viscoelastic limits extending to strains of approximately 0.1–0.3%. This behavior is consistent with reports in the literature for neat polymers [46,47,48,49]. The storage modulus (G′) remained independent of the strain at low stress. However, at higher strain levels, a sudden drop in G′ indicated structural deformation, signifying a transition from elastic to viscous behavior and the disentanglement of macromolecules [50,51]. Similar studies have demonstrated that incorporating PHB in binary blends decreases the storage modulus [49]. In contrast, combining neat PLA and PBAT significantly increases storage modulus across all strain rates compared to the pure polymers [52].

**Figure 2:**
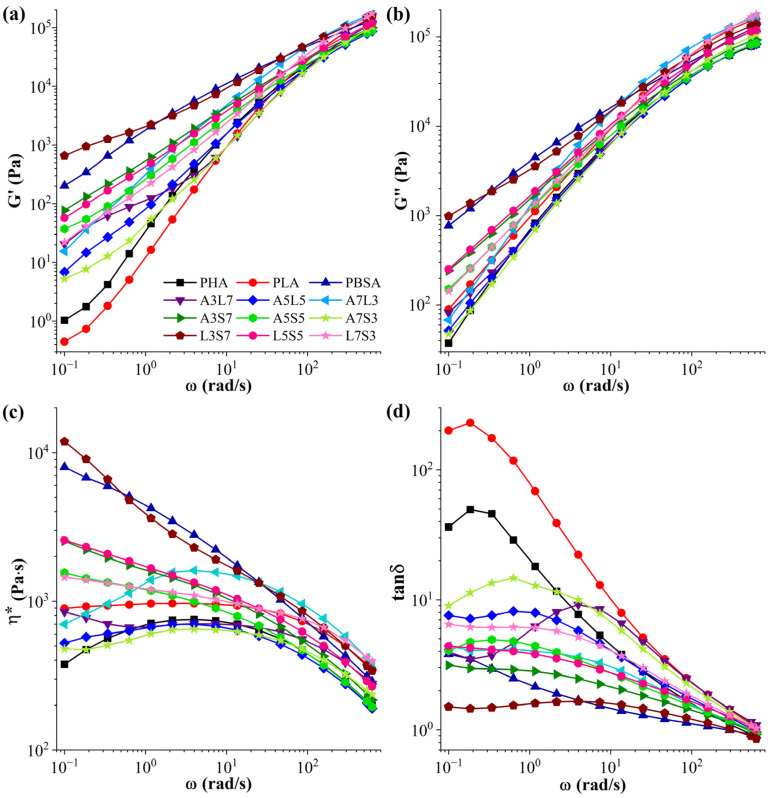
Frequency dependence of (**a**) storage moduli (G′), (**b**) loss moduli (G″), (**c**) complex viscosity (η*), and (**d**) loss factor (tanδ) for binary PHA/PLA, PHA/PBSA, and PLA/PBSA blends at 180 °C.

Figure 2 presents the storage (G′) and loss (G″) moduli, complex viscosity (η*), and loss factor (tanδ) values as functions of angular frequency (*ω*) for the binary blends of PHA, PLA, and PBSA. All blends exhibited a typical increase in both G′ and G″, with an increase in ω (Figure 2a,b). L3S7 displayed the highest values among the blends, while L7S3 showed the lowest across all frequency ranges. G′ at low frequencies was related to the shape change of the dispersed phase in the matrix during the oscillatory test [53,54]. In particular, the increase in G′ at low frequencies for immiscible blends was associated with the relaxation and deformation time of the deformed dispersed phase and became more noticeable for large particle sizes [55,56].

The most notable increase in G′ was observed for pure PHA and PLA, while all blends exhibited significantly higher storage moduli than pure polymers. At higher frequencies, the incorporation of PBSA had a minimal effect on the blends and G″. However, PBSA increased the storage and loss moduli at lower frequencies. According to the literature, an increase in the number of entanglements consistently raises the viscosity of the polymer blends [57,58].

As displayed in Figure 2c, PLA exhibited Newtonian behavior within the frequency range of 0.1–10 rad/s, a region also referred to as zero shear. Conversely, the remaining polymers and blends displayed non-Newtonian behavior across the entire frequency range, which is usually associated with a molecular weight distribution [59]. As reported in the literature, neat PHA degrades over time at high temperatures, near or above its melting point. This degradation results in a drop in viscosity at low frequencies (below 1 rad/s) [60,61,62,63,64]. The measurement was set up to run from higher to lower frequency values; the viscosity of neat PHA and A7L3, A7S3, and A5L5 blends decreased at lower frequencies as the residence time in the rheometer exceeded the stability of the polymer. The viscosity value primarily depended on the molecular weight of a polymer and on the morphology of the polymer blends. The dispersion and the sizes of the dispersion phases (Figure S1) can induce pronounced differences in rheological properties. The changes in viscosity values of binary blends through a variation in the composition of the matrix phase induced droplet break-up at the dispersed phase. Similar results have been reported in the literature [65,66,67].

Blending PBSA with PHA or PLA can improve the shear flow, increase the viscosity, and avoid the degradation process of the blends [68,69]. Typically, a higher viscosity at low frequencies helps maintain shape integrity during extrusion. Conversely, at high frequencies, a lower viscosity is advantageous for achieving optimal conditions in injection molding processes [70,71].

The relaxation process of viscoelastic materials is characterized by the loss tangent (tanδ), which reflects the mobility of polymer segments and chains. A peak in tanδ, observable in the A5L5, A3L7, and A7S3 blends as shown in Figure 2d, indicates a transition to a different microstructure. A larger tanδ (tanδ = G″/G′) represents a more viscous material behavior and higher energy loss, whereas a lower tanδ indicates more elastic behavior [72]. For the blends, the tanδ values were lower than those of neat PHA and PLA but higher than those of neat PBSA. Among the blends, A7S3 exhibited the highest tanδ value, while L3S7 had the lowest. The data indicated that the tanδ of the binary blends decreased with increasing PBSA content, suggesting that the addition of PBSA improved the elasticity of the blends, due to PBSA’s higher viscosity compared to PHA and PLA [72].

**Figure 3:**
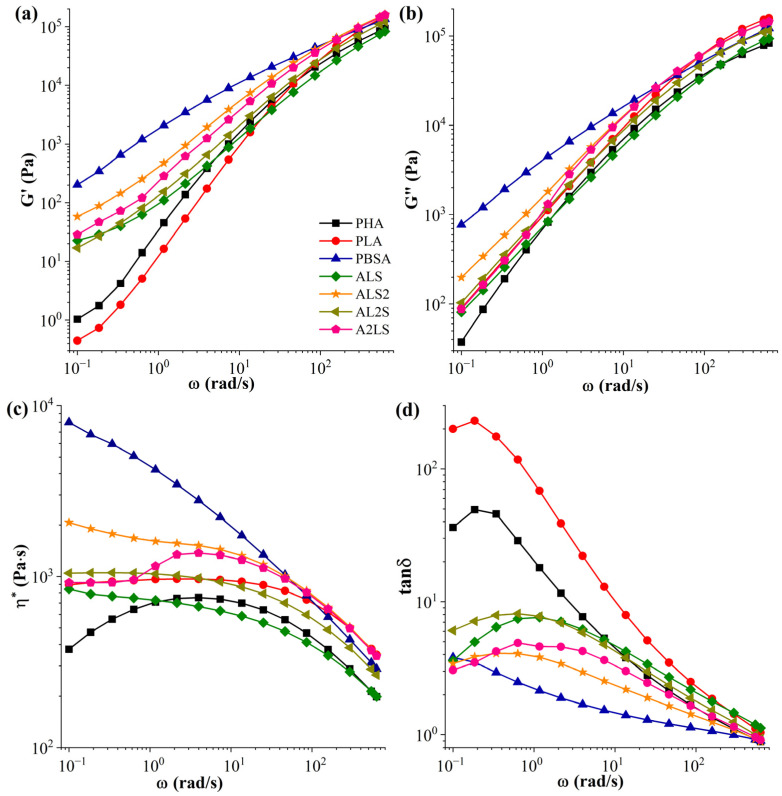
Frequency dependence of (**a**) storage moduli (G′), (**b**) loss moduli (G″), (**c**) complex viscosity (η*), and (**d**) loss factor (tanδ) for ternary PHA/PLA/PBSA blends at 180 °C.

Figure 3 illustrates the G′ and G″ moduli, η*, and tanδ as functions of ω for the ternary PHA/PLA/PBSA blends. The blend ALS2 exhibited the highest G′ and G″ values (Figure 3a,b), which can be attributed to the impact of PBSA. In contrast, the blend ALS showed the lowest G′ and G″ across all frequency ranges. The AL2S blend demonstrated Newtonian behavior within the frequency range of 0.1–10 rad/s (Figure 3c). In contrast, the A2LS blend exhibited the PHA degradation discussed above. Furthermore, AL2S exhibited a viscosity behavior similar to PLA. The presence of PBSA in ALS and AL2S adversely influenced the blend’s processing properties, indicating a comparatively weak interface. This can substantially impact the mechanical properties of the blends. Conversely, for the ALS2 blend, PBSA appeared to benefit the blend’s properties and thermal processing. Unlike pure polymers, blends exhibit an elastic response at low frequencies, attributed to the macromolecular chain orientation stemming from the developed blend morphology [12]. However, owing to macromolecular slippage at high frequencies, all blends and neat polymers displayed similar behavior. In Figure 3d, the highest tanδ can be observed for AL2S and ALS blends. The tanδ gradually rose with the increasing PLA and PHA concentrations. With the introduction of PBSA, the elasticity of the blends improved. Such improvements can commonly be observed with branching and cross-linking, but the authors are unaware of such structural elements in the PBSA structure [73]. It has been reported that incorporating varying amounts of PHBHH and PBS into PLA/PBS/PHBHH blends enhances the viscoelastic properties and eliminates the Newtonian plateau in the low-frequency region [10,73]. This elimination is attributed to the interactions at the interface between the different components. Qiao et al. noted that PLA and PHBV, being similar linear polymers, exhibit comparable rheological characteristics, particularly at high frequencies [74]. However, due to the greater chain regularity in PHBV, it had lower viscosity values than PLA, despite PHBV’s higher molecular weight.

To describe the melt viscosity and quantity of the shear thinning of polymeric materials, the power law model is usually used (Equation (2)) [61,75,76], as follows:

(2) $$\eta =K{\left|\gamma^{.}\right|}^{n-1}$$

where η is a viscosity (Pa∙s), K is the consistency (Pa∙s^n^), $\gamma^{.}$ is a shear rate (s^−1^), and n is the shear thinning index. The n≠1 describes the rheology of non-Newtonian fluids, while n=1 represents Newtonian fluids [77,78].

Power law model parameters such as shear thinning index (n), consistency parameter (K), and zero shear viscosity ($\eta_{0}$) are shown in Table 2. The index n varied from 0.540 to 0.675. All samples exhibited non-Newtonian fluid and pseudoplastic behavior. A7S3 and ALS achieved the highest shear thinning index values for binary and ternary blends, respectively. Increasing the amount of PHA and PLA in the blends raised the index n value and the Newtonian behavior of binary and ternary blends. Multiple research investigations have established correlations between the polydispersity of PBSA, the distribution of its molecular chains, and the resultant impact on chain scission phenomena [76,79,80]. These factors significantly affect the behavior of the shear thinning index. Additionally, the variations in the n index can be related to macromolecular entanglement and chain orientation in the blends [81]. The consistency parameter (K) matches the value of n [12]. The parameter K for PLA/PBSA and PHA/PBSA blends rose with increasing PBSA concentration. The highest K value was 3.808 for the A7L3 binary blend. When the viscosity is constant at low frequencies, it is known as the zero-shear or Newtonian viscosity [61]. The lowest $\eta_{0}$ values were 647 Pa∙s and 747 Pa∙s for binary A7S3 and ternary ALS blends, respectively. However, binary A3S7 and ternary ALS2 blends demonstrated the highest $\eta_{0}$ values, which were 9051 Pa∙s and 1671 Pa∙s, respectively. Different values of zero-shear viscosity can be related to variations in polymer molecular weight and entanglement [61].

**Figure 4:**
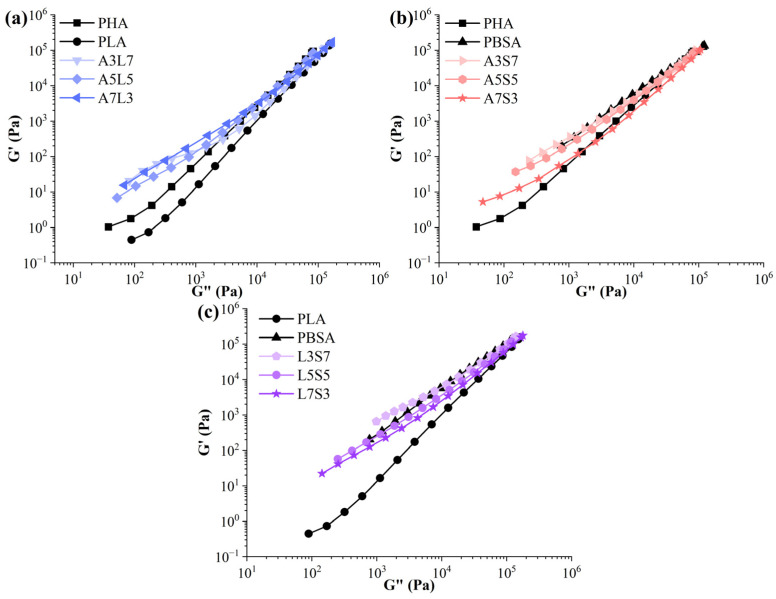
Han plot diagram giving the storage modulus (G′) versus loss modulus (G″) for the (**a**) PLA/PHA, (**b**) PHA/PBSA, and (**c**) PLA/PBSA binary blends.

The Han plot, which depicts a linear relationship in the plot of logG′ versus logG″, was utilized to examine the miscibility of binary blends of PHA, PLA, and PBSA through rheological data (Figure 4) [60,82]. Compatible blends have the same slope for different compositions of the polymer components. Otherwise, the blends are considered to be immiscible [83,84]. The analysis revealed that all PHA/PBSA and PLA/PBSA blends displayed varying slopes and a nonlinear correlation, indicating poor miscibility between the components [85]. In contrast, the Han plot for PHA/PLA blends exhibited partial compatibility [71]. The minor variations observed in the terminal regions of the curves can be attributed to the polydispersity of the individual polymer components and the introduced microheterogeneity [86]. From a rheology perspective, a degree of miscibility between PHA and PLA has been demonstrated, attributed to the polymers’ similar linear structures [86].

### 3.2. Calorimetric Properties

**Figure 5:**
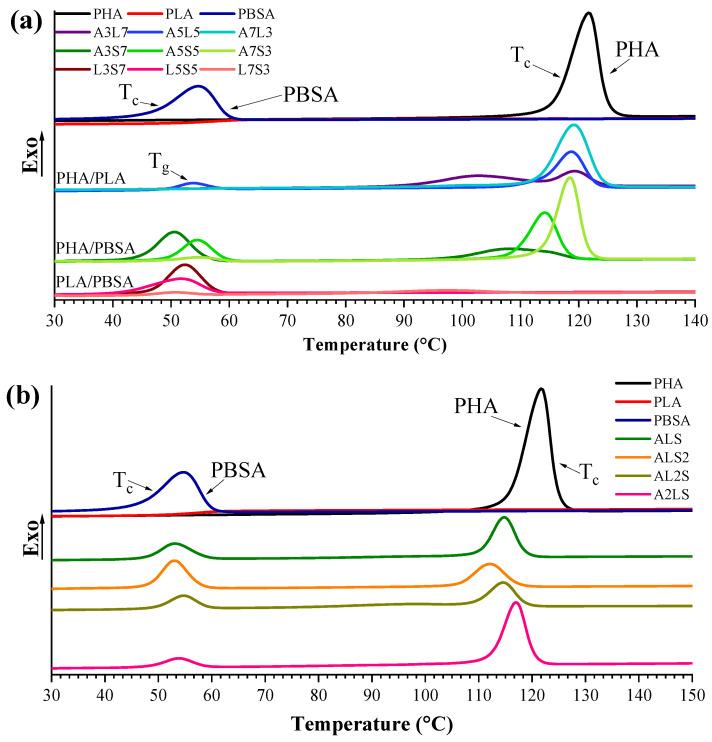
DSC cooling scans for neat PHA, PLA, and PBSA polymers and their (**a**) binary and (**b**) ternary blends with a cooling rate of 10 °C min^−1^.

**Figure 6:**
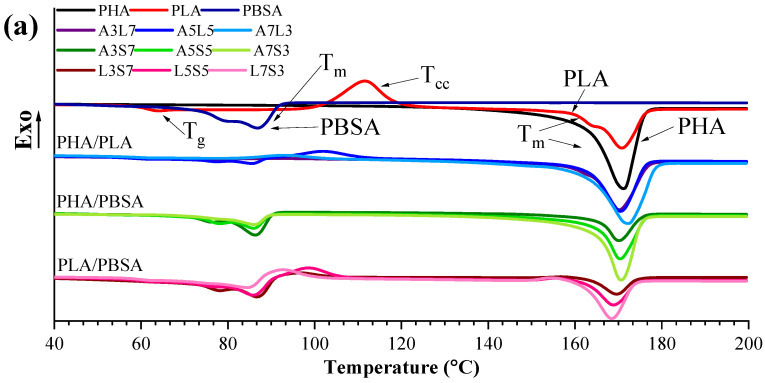

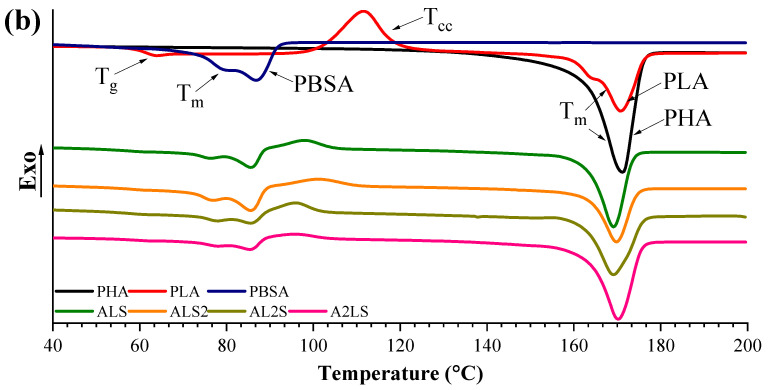
DSC second heating scans for neat PHA, PLA, and PBSA polymer and their (**a**) binary and (**b**) ternary blends with the heating rate of 10 °C min^−1^.

The thermal properties of the binary and ternary PHA, PLA, and PBSA blends can be significantly affected by the crystallization characteristics of individual polymers. Figure 5 and Figure 6 show DSC cooling and second heating scans for binary and ternary blends. The related crystallization and melting properties for binary and ternary blends are listed in Table 3 and Table 4, respectively. These include crystallinity (χ%), melting (T_m_), crystallization (T_c_), and glass transition (T_g_) temperatures. Moreover, the measurements can be used to determine the immiscibility of polymer blends. A single temperature for phase transition can be observed if a blend consists of compatible polymers. In the case of incompatible polymers, two or more T_c_ and T_m_ are visible in the DSC curve.

The cooling scans show two crystallization peaks for all ternary and binary PHA/PBSA blends. The crystallization rate of PLA was relatively slow, resulting in the absence of an exothermic peak, which was attributed to its amorphous structure (and lack of nucleating fillers) [81,87]. However, pronounced exothermic peaks were observed for PHA and PBSA. The crystallization peak of neat PHA was visible at 122 °C, whereas the T_c_ of neat PBSA was about 55 °C.

As seen in Table 3 and Figure 5a, the A3L7 blend exhibited two crystallization peaks at 102.9 °C and 119.3 °C, attributed to PLA and PHA, respectively. In blends containing PLA, the PHA crystallization temperature was slightly shifted to lower temperatures. In contrast, a notable shift was observed in PHA/PBSA blends. For instance, A3S7 showed one broad peak for PHA, which shifted by up to 14 °C compared to neat PHA. This reduction was attributed to phase restriction and nucleation suppression induced by PLA and PBSA [88,89].

PLA predominantly maintained its amorphous structure across the blends, with minor exceptions in the L7S3 and A3L7 compositions. The crystallinity of PLA in A3L7 was 35%, while in L7S3, the calculated crystallinity was modest, totaling only 8%. This indicates that PHA promoted the crystallization of PLA more effectively than PBSA.

The second heating scan showed two melting peaks and cold crystallization (T_cc_) processes for both binary and ternary blends. When PLA does not crystallize during cooling, its T_cc_ occurs around 110 °C, which can be observed during a heating scan [23]. The melting enthalpy of PLA corresponded to its cold crystallization and was of similar value. For binary PHA/PLA blends, the melting enthalpy closely matched the sum of the crystallization enthalpy of PHA and the cold crystallization enthalpy of PLA. PLA’s cold crystallization peak was observed for all blends that contain PLA. Compared with neat PLA, the T_cc_ of the PLA in the blends was shifted to a lower temperature, implying that the PHA and PBSA accelerated the cold crystallization. The enhancement of the T_cc_ of PLA was attributed to activated chain mobility. PHA and PBSA amorphous phases were locally activated, and T_cc_ was improved due to the quick dynamic alignment of the chain. PHA and PBSA’s domains might have acted as nucleating centers and may have accelerated the crystallization behavior of PLA [90,91]. Also, it should be noted that the T_m_ of PBSA was below the T_cc_ of PLA. Like binary blends, the T_cc_ of PLA in ternary blends was shifted, forming PLA’s crystallites at lower temperatures because of PHA and PBSA [92].

The T_g_ can be observed for neat PLA at 60 °C, but it was not pronounced in all binary and ternary blends due to its overlap with the T_m_ of PBSA. PHA/PBSA blends showed melting peaks around 170 °C (PHA) and 85–86 °C (PBSA), and PLA/PBSA around 168–169 °C (PLA) and 84–86 °C (PBSA). Furthermore, only one endothermic peak at 170 °C for PHA/PLA blends was observed, which saw a slight 3 °C increase in the A7L3 blend. The overlap in PHA and PLA’s melting peaks can be related to similar macromolecular interaction types, and their blends may be partially miscible [93]. Still, this overlap may also have been a coincidence. PBSA polymer and its blends showed two melting peaks or a single peak with a shoulder at 84–86 °C. It is suggested that PBSA can form two different crystallization structures with disordered (imperfect) crystallites. The peak at the lowest temperature represents the melting of the disordered crystalline structure, while the peak at a higher temperature represents the ordered crystalline form, which is more stable and requires a higher melting temperature [7,94,95]. During the melting of disordered crystalline structure, recrystallization can occur, but it was not observed as a visible peak in this study. The T_m_ of PBSA in ternary blends varied from 68 to 86 °C, while, for PHA and PLA, the melting peaks were around 169–170 °C. The fusion of PHA and PLA melting peaks occurred, making it hard to distinguish each polymer’s melting peak.

### 3.3. Ternary Blends’ Morphology and Compatibility

The morphology of PHA, PLA, and PBSA binary blends has been extensively discussed in the literature, and studies on the morphology of ternary biopolymer blends are scarce. Our previous research has indicated that binary PBS/PLA blends exhibit better compatibility with lower concentrations of PBS [12]. Additionally, we observed that PLA forms smaller and more uniformly distributed spherical inclusions within the PBS matrix compared to PHB. This finding aligns with other studies, which demonstrate that PBS/PHB blends consistently display rough surfaces characterized by larger, variably sized spherical inclusions with irregular distribution [95,96], thus forming larger domains of individual components. It has been reported that PLA/PHBV blends comprising high molecular weight polymers exhibit a biphasic structure along with two glass transition temperatures, indicating the polymers’ immiscibility [97]. Gerard et al. reported that PLA/PHA blends present a nodular structure when one of the polymers constitutes less than 30 wt.% and a co-continuous structure when the polymer proportions are equal [63].

**Figure 7:**
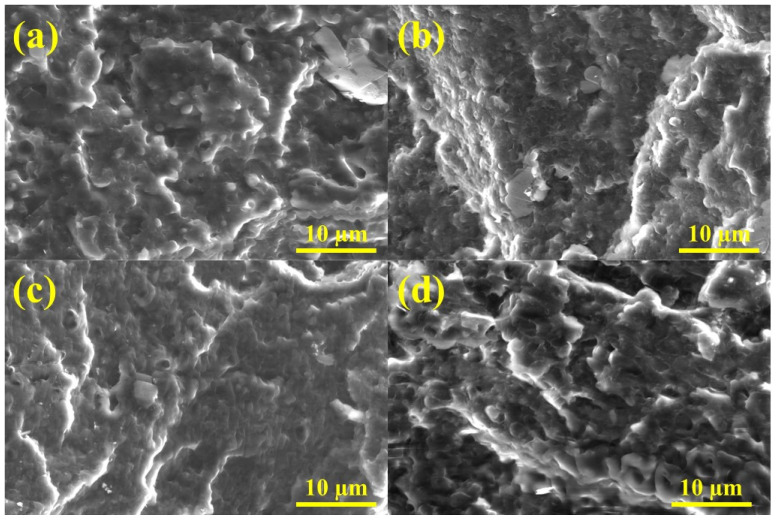
SEM images of cross-section morphologies produced by liquid nitrogen fracture of injection molded rods: (**a**) A2LS, (**b**) AL2S, (**c**) ALS2, and (**d**) ALS ternary blends.

The compatibility and the impact of ratio variations in binary and ternary blends of polymers were assessed by investigating their morphology. SEM micrographs of the cryo-fractured surfaces of PHA/PLA/PBSA binary blends are shown in Figure S1, while those for ternary blends are presented in Figure 7 and Figure S2. Binary blends with three/seven and seven/three ratios exhibited various forms of spherical inclusions representing the minority component. As discussed earlier, PHA in the form of spherical inclusions was characterized by larger, variably sized droplets with irregular distribution. In contrast, PLA spherical inclusions were the hardest to distinguish in the binary blend images. The five/five ratio presented three distinct morphologies, as follows: A5S5 showed pronounced large irregular domains of individual components, indicating poor compatibility for this composition. The A5L5 blend appeared to form the most uniform structure, while L5S5 presented a layered structure. Interestingly, although the domains in L5S5 appeared larger with a distinct interface, no large pull-outs were visible, and the surface appeared smoother than that of A5S5.

All ternary blends exhibited similar, to some extent, droplet morphologies, accompanied by visible pull-out voids. This observation strongly indicates the immiscibility of at least two blended polymers. The continuous formation of pronounced droplets was particularly noticeable in the ALS blend, likely due to the absence of a predominant component. All ternary blends exhibited more heterogeneous structures and rougher fracture surfaces compared to binary blends. This could be attributed to the majority component not exceeding 50 wt.%. In the literature, the blend incompatibility has been linked to the crystalline structure of PHB and its degradation during thermal and mechanical processing [71].

## 4. Conclusions

Three bio-based and biodegradable polymers, PHA, PLA, and PBSA, were successfully mixed in binary and ternary blends using the melt extrusion method. The examined properties of these blends revealed a unique set of tunable thermal and rheological properties that the individual polymers lack. Compared to pure polymers, the blends exhibited elastic responses at lower frequencies. The addition of PBSA enhanced the viscoelastic behavior of PLA and PHA in the binary and the ternary blends. However, the addition of PBSA to ternary blends negatively impacted processing properties. ALS2’s high viscosity ratio led to irregular structure morphologies and could further account for the maximum degree of immiscibility, while AL2S showed better processing properties due to a higher amount of PLA. A Han diagram was used to investigate the morphology of binary blends at the melt state. The results showed that PHA/PBSA and PLA/PBSA blends were immiscible in the melt state. However, PHA/PLA showed partial blend miscibility at low-frequency ranges correlated with DSC measurements. The results demonstrate the possibility of producing biopolyester-based materials with well-balanced thermal and calorimetric properties as an alternative to conventional commodity plastics. SEM images showed that the blend morphology benefited from a reduced PHA content. The fundamental lack of miscibility among binary and ternary biopolymer blends requires the introduction of compatibilizers to realize their full potential, optimizing blend morphology and interfacial interactions to achieve synergistic properties. Future research could be directed towards improvements in the rheological and thermal properties of the blends by using appropriate bio-based additives. This strategic approach could enhance biopolymer blends’ performance and application range, aligning them more closely with the requirements of various end-use products and contributing to developing more sustainable material solutions.

## Figures and Tables

**Table 1:** Abbreviations and formulations.

Sample	PHA (A) (wt.%)	PLA (L) (wt.%)	PBSA (S) (wt.%)
PHA	100	0	0
PLA	0	100	0
PBSA	0	0	100
A3L7	30.0	70.0	0
A5L5	50.0	50.0	0
A7L3	70.0	30.0	0
A3S7	30.0	0	70.0
A5S5	50.0	0	50.0
A7S3	70.0	0	30.0
L3S7	0	30.0	70.0
L5S5	0	50.0	50.0
L7S3	0	70.0	30.0
ALS	33.3	33.3	33.3
ALS2	25.0	25.0	50.0
AL2S	25.0	50.0	25.0
A2LS	50.0	25.0	25.0

**Table 2:** Power law model parameters.

Samples	n	$K\;(Pa\cdot s^{n}$)	$\eta_{0}$ (Pa·s)
PHA	0.597	3.440	753
PLA	0.625	3.606	966
PBSA	0.591	3.659	5954
A3L7	0.651	3.432	702
A5L5	0.615	3.374	699
A7L3	0.568	3.808	1610
A3S7	0.540	3.633	2537
A5S5	0.559	3.532	1554
A7S3	0.675	3.297	648
L3S7	0.553	3.791	9051
L5S5	0.600	3.563	2570
L7S3	0.661	3.555	1193
ALS	0.660	3.265	747
ALS2	0.588	3.707	1671
AL2S	0.624	3.495	1036
A2LS	0.595	3.682	1371

**Table 3:** Thermal properties of binary PHA, PLA, and PBSA blends.

Samples	Component	T_c_ (°C)	ΔH_c_ (J/g)	χ_c_ (%)	T_m_ (°C)	ΔH_m_ (J/g)	T_c_ (°C)	ΔH_c_ (J/g)
PHA		121.7	84.0	58	171.2	92.9		
PLA		–	–	–	170.7	41.9	111.6	42.1
PBSA		54.6	44.0	40	86.8	27.4		
A3L7	PHA	119.3 **	11.3	26	170.1	50.9		
	PLA	102.9 **	22.6	35	170.1 *	50.9 *	91.7	0.4
A5L5	PHA	118.8	31.8	44	170.6	51.9		
	PLA	–	–	–	170.6 *	51.9 *	101.78	10.8
A7L3	PHA	119.1	60.8	60	173.2	69.2		
	PLA	–	–	–	173.2 *	69.2 *	92.9	3.8
A3S7	PHA	108.1	20.8	48	170.2	22.0		
	PBSA	50.4	29.3	38	86.3	20.89		
A5S5	PHA	114.2	38.8	53	170.5	40.8		
	PBSA	54.6	19.2	35	85.8	13.6		
A7S3	PHA	118.5	56.3	55	170.6	62.8		
	PBSA	54.9	4.6	14	86.0	10.9		
L3S7	PLA	–	–	–	169.6	12.5	96.8	4.2
	PBSA	52.4	26.4	34	86.6	36.8		
L5S5	PLA	–	–	–	169.0	19.7	98.6	18.1
	PBSA	51.9	18.1	33	85.8	23.4		
L7S3	PLA	97.5	5.1	8	168.4	29.6	95.8	4.2
	PBSA	50.8	1.8	5	84.4	14.0		

* PHA and PLA melting peaks overlay. ** PHA and PLA crystallization peaks overlay.

**Table 4:** Thermal properties of ternary PHA, PLA, and PBSA blends.

Samples	Component	T_c_ (°C)	ΔH_c_ (J/g)	χ_c_ (%)	T_m_ (°C)	ΔH_m_ (J/g)	T_c_ (°C)	ΔH_c_ (J/g)
ALS	PHA	114.7	23.4	49	169.1	35.3		
	PLA	–	–	–	169.1 *	35.3 *	98.1	6.8
	PBSA	53.2	11.7	32	76.2	0.3		
ALS2	PHA	112.1	18.4	51	169.9	31.9		
	PLA	–	–	–	169.9 *	31.9 *	101.1	8.1
	PBSA	53.1	19.5	36	85.6	17.3		
AL2S	PHA	114.6	14.0	38	170.1	38.6		
	PLA	–	–	–	170.1 *	38.6 *	96.8	15.0
	PBSA	54.8	8.2	30	68.7	8.2		
A2LS	PHA	117.1	38.	52	170.2	43.5		
	PLA	–	–	–	170.2 *	43.5 *	95.8	4.2
	PBSA	53.8	5.9	22	85.2	0.9		

* PHA and PLA peaks overlay.

## Data Availability

Data will be made available on request.

## Supplementary Materials

The following supporting information can be downloaded at: https://www.mdpi.com/article/10.3390/polym16162288/s1, Figure S1. SEM images of cross-section morphologies of binary blends. Figure S2. SEM images of cross-section morphologies of ternary blends.
