# Supplementary material for: Exploring the Processing Potential of Polylactic Acid, Polyhydroxyalkanoate, and Poly(butylene succinate-co-adipate) Binary and Ternary Blends
Source: Polymers (Basel). 2024 Aug 13;16(16):2288. doi: 10.3390/polym16162288 (PMC11360580; doi:10.3390/polym16162288)
Supplement: Supplementary file 1 [file polymers-16-02288-s001.zip › polymers-3099542-supplementary.pdf]

Supplementary Materials

Exploring the Processing Potential of Polylactic Acid, Polyhydroxyalkanoate, and Poly(butylene succinate-*co*-adipate) Binary and Ternary Blends

Alisa Sabalina [1,*], Sergejs Gaidukovs [1,*], Arturs Aunins [1], Anda Gromova [1], Gerda Gaidukova [1], Liga Orlova [2] and Oskars Platnieks [1]

[1]Institute of Chemistry and Chemical Technology, Faculty of Natural Sciences and Technology, Riga Technical University, P. Valdena 3, LV–1048 Riga, Latvia; arturs.aunins@edu.rtu.lv (A.A.); anda.gromova@rtu.lv (A.G.); gerda.gaidukova@rtu.lv (G.G.); oskars.platnieks_1@rtu.lv (O.P.)
[2]Institute of Materials and Surface Engineering, Faculty of Natural Sciences and Technology, Riga Technical University, P. Valdena 3, LV–1048 Riga, Latvia; liga.orlova@rtu.lv (L.O.)
*Correspondence: alisa.sabalina@rtu.lv (A.S.); sergejs.gaidukovs@rtu.lv (S.G.)

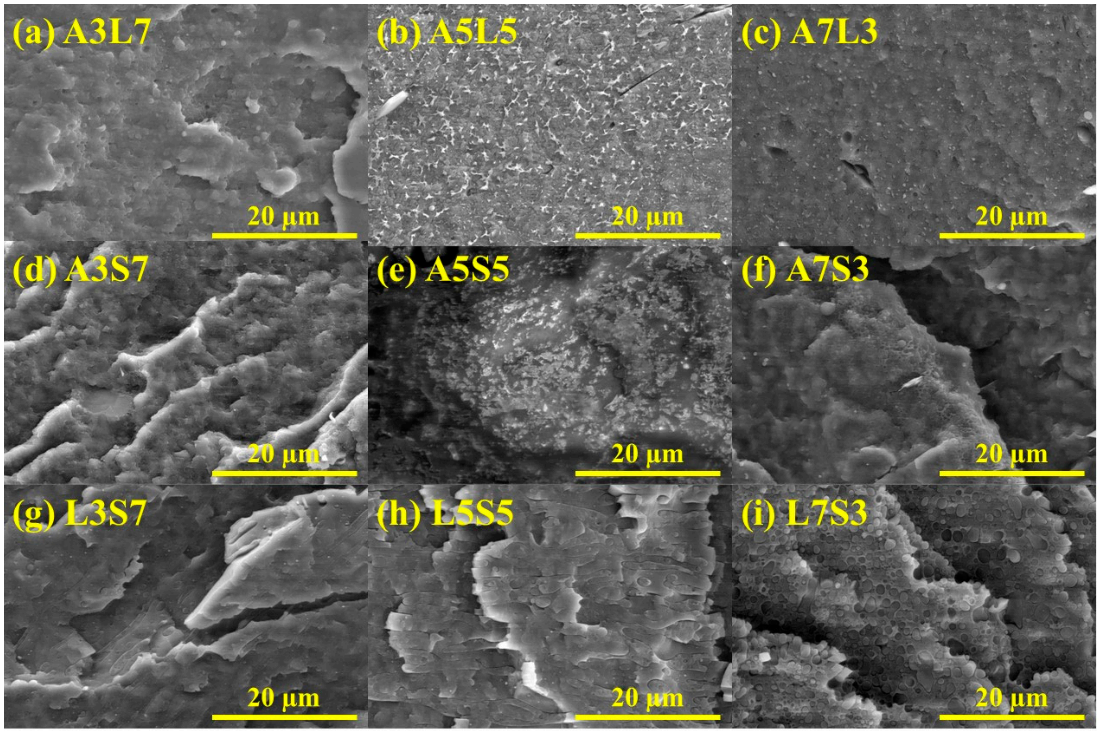

Figure S1. SEM images of cross-section morphologies produced by liquid nitrogen fracture of injection molded rods (a)-(c) PHA/PLA, (d)-(f) PHA/PBSA, and (g)-(i) PLA/PBSA binary blends.

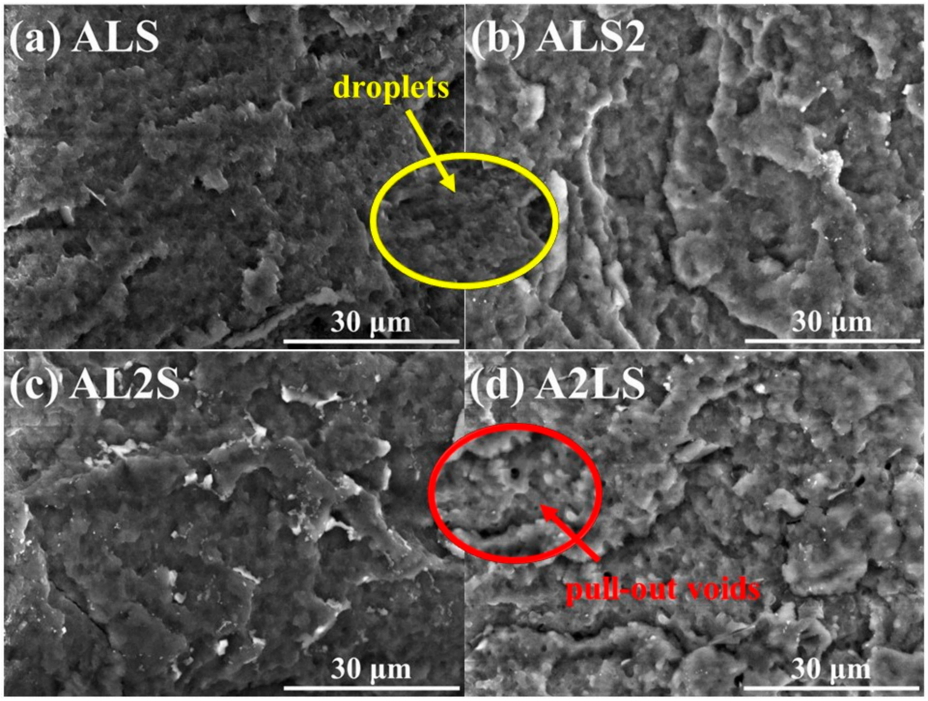

Figure S2. SEM images of cross-section morphologies produced by liquid nitrogen fracture of injection molded rods (a) ALS, (b) ALS2 (c) AL2S, and (d) A2LS ternary blends.
